# Consumer Misuse of Country-of-Origin Label: Insights from the Italian Extra-Virgin Olive Oil Market

**DOI:** 10.3390/nu12072150

**Published:** 2020-07-19

**Authors:** Francesco Bimbo, Luigi Roselli, Domenico Carlucci, Bernardo Corrado de Gennaro

**Affiliations:** Department of Agricultural and Environmental Sciences, University of Bari Aldo Moro, 70126 Bari, Italy; francesco.bimbo@uniba.it (F.B.); domenico.carlucci@uniba.it (D.C.); bernardocorrado.degennaro@uniba.it (B.C.d.G.)

**Keywords:** consumer choice, food labeling, extra virgin olive oil, hedonic price model, country of origin

## Abstract

Providing information to consumers through the label is a means for food companies to inform consumers about product’s attributes, including the country of origin (COO). In the EU, COO labeling has been made mandatory for several categories of food products, to enable consumers to make informed choices at the point of sale. In particular, Regulation (EU) No 29/2012 has introduced a mandatory country-of-origin labeling system for extra virgin olive oil (EVOO). In the present study, conducted in Italy, we test whether there is a price differential associated with the COO information for EVOO. To this end, we employ a hedonic price model and data about the purchase of EVOO products collected from 982 consumers at the supermarket checkout. Having interviewed these consumers, we also assess the share of EVOO consumers that correctly identify the country of origin of the product purchased. Our findings point out that, in Italy, the EVOO with domestic origin, indicated on the label, benefits of a premium price equal to +35% compared to the product labeled as blend of European EVOOs, while a discount of −10.8% is attached to EVOOs from a non-European origin. A significant share of consumers in our sample (19.04%) is, however, unable to correctly identify the origin of the EVOO purchased. This label misuse mostly occurs among consumers who report that they had purchased Italian EVOO, while they had actually purchased a blend of European EVOOs. Female and more highly educated consumers are less likely to misuse label information about the product’s origins.

## 1. Introduction

Extra-virgin olive oil (EVOO) is the superior olive oil category extracted from olives by the mechanical extraction process. EVOO is one of the main components of the Mediterranean diet and it is considered worldwide as one of the healthiest oils. The European Union (EU) is the largest EVOO producer worldwide, and production is concentrated in three Mediterranean countries: Spain produces about 57.5% of EU EVOO output, Italy over 19.5%, and Greece about the 15.8% [1]. Due to increasing consumption in non-producing countries, both within the EU (e.g., UK and Germany) and outside Europe (e.g., China, Japan, Russia, Australia, Brazil, Canada, and the US), demand for European EVOO is expected to rise until 2030 [2,3]. Rising EVOO consumption in non-producing countries leads Mediterranean EVOO producers to export their EVOO to other markets [2,3]. The steadily increasing demand for EVOO in non-producing countries has led Italy to export a large share of domestically produced EVOO, which is no longer sufficient to satisfy the domestic market. Such supply imbalance is constantly re-balanced by importing EVOO from Spain and non-European Mediterranean countries, such as Tunisia and Turkey. This EVOO is usually priced lower than Italian EVOO. Compared to Italian EVOO producers, the Spanish benefit from economies of scale and non-European producers with lower labor costs [3]. Production costs at farm level for a liter of Italian EVOO are, on average, 30% higher than the production costs recorded for Spanish and non-European Mediterranean producers [1]. As a result, Italian EVOO sold on the Italian market competes with EVOO imported from other countries.

Over the last three decades, consumers have placed increasing importance on the product’s origin. However, the latter cannot be verified either ex-ante or ex-post consumption and asymmetric information about the products’ origins arises between producers and consumers. Providing information about products’ origins through the label has become a widely adopted tool to mitigate the information gap between consumers and producers. Country of origin labeling policies, by informing consumers about a product’s origin, transform information about the origin of the product, that is a credence attribute, into a searchable characteristic and so alleviate the problem of asymmetric information [4,5] Generally speaking, labeling policies address a market failure, asymmetric information, through costly expenditures borne by a combination of consumers, firms, and taxpayers. First, the industry bear costs of labeling, which likely pass on consumers at higher prices. Second, the government’s costs of label monitoring and enforcement system are borne by taxpayers via higher taxes. Third, mandatory label exacerbates other market distortions such as decrease competition or encourage rent-seeking and gaming, as well as introduces trade distortions across countries [6]. 

In the case of the EVOO market, EU policymakers have introduced Regulation (EU) No 29/2012, a mandatory labeling information system requiring producers to indicate on the label the country of origin (COO) of the EVOO. This Regulation establishes that the labeling of extra virgin olive oil and virgin olive oil must bear a designation of origin. Bottlers can use, on the label, one of the following claims relating to origin: (a) in the case of olive oils originating from one Member State or third country, a reference to the Member State, to the Union, or to the third country, as appropriate; (b) in the case of blends of olive oils originating from more than one Member State or third country; or one of the following statements, as appropriate: (i) ‘blend of olive oils of European Union origin’ or a reference to the Union; (ii) ‘blend of olive oils not of European Union origin’ or a reference to origin outside the Union; (iii) ‘blend of olive oils of European Union origin and not of European Union origin’ or a reference to origin within the Union and outside the Union; or (c) a protected designation of origin or a protected geographical indication referred to in Regulation (EU) No 1151/2012, in accordance with the provisions relating to the product specification concerned [7]). 

On the one hand, the introduction of Regulation No (EU) 29/2012, by informing consumers about the origin of EVOO, is potentially beneficial to Italian EVOO producers. Since Italian consumers strongly prefer domestic EVOO over non-Italian alternatives and are willing to pay a premium price for it [3,8,9,10,11], the regulation would support Italian producers to ensure fair revenues for their product. In other words, by informing consumers about the COO of EVOO, Italian producers are able to differentiate their products and add value to these.

On the other hand, the measures based on informing consumers through the label implicitly assume that information on the label eliminates asymmetric information by fully informing consumers about the product’s features, and so restoring information symmetry between consumers and producers. The existing literature points, however, to many instances where the labeling policy is not be able to restore full information. This occurs because consumers either do not make full use of the label, or the label’s information is not clear, or consumers are not fully aware of the information’s availability [12]. Furthermore, consumers may not fully trust label information due to the risk of incurring food fraud. Frauds are more likely to occur in products that benefit from premium prices. Indeed, the latter work as an incentive for producers to commit fraud [13].

As a result, the aim of the present study is twofold: (i) to test whether there is a price differential associated with COO information for EVOO by using retail level data collected from Italian EVOO consumers purchases; and, (ii) to assess in what measure consumers correctly use the information about the origin of the EVOO they purchased. To the best of our knowledge, no previous studies investigated to what extent consumers correctly identify the origin of the EVOO purchased by using the origin information on the label. We did so by interviewing EVOO shoppers at the supermarket checkout about the origins of the product they have purchased. By inspecting the labeling of the product these shoppers purchased, we assess whether their understanding of the product’s origin matches what is reported on the label. We then analyze consumer groups of differing abilities in identifying the COO of the product according to their socio-economic characteristics, as well as their self-declared interest in the product’s label information, the origin of the product, and interest in branded products more widely. Indirectly, this allows us to infer the effectiveness of the mandatory COO label in correctly informing consumers and orienting their food choices. The remainder of the paper is structured as follows: the next section presents a description of survey design, the data and the model used; then, we discuss the empirical results. We conclude by providing recommendations for EVOO producers and policymakers.

## 2. Materials and Methods 

### 2.1. Survey and Data Collection

The survey involved 982 EVOO consumers interviewed at the supermarket checkout counters. Consumers were selected on voluntary basis and did not receive any monetary compensation to participate in the study. Once consumers were approached at supermarket cashiers were asked their willingness to participate in the study or not. For those who accepted to participate, written informed consent according to the national ethical requirement “Italian Personal Data Protection Code” (L.D. 196/2003) was collected. Then, the interviewers asked them to state the origin of the EVOO they had just purchased. Consumers were free to answer by selecting one of the following statements: “I am unaware of the origin of the EVOO I purchased”, “It is a blend of non-European EVOOs”, “It is a blend of European EVOOs” and “It is a 100% Italian EVOO”. The interviewers then inspected the labeling of the product purchased. By comparing consumers’ answers and the information on the origin of the EVOO found on the label, they were able to assess the correctness of what consumers stated about the COO of the EVOO purchased. The share of consumers that correctly identified the origins of the product was used as a proxy for the effectiveness of the EU Reg. 29/2012 to inform consumers about such origins. 

The interviewers also collected information on the characteristics of the EVOO that consumers purchased, including its COO attribute, used for sizing their monetary value by means of the hedonic price model. Socio-economic information about the consumers, if they shopped for EVOO as a result of price promotion, as well as their interest in labeling information, in the origin of products, and preference for branded products, were also collected. These served as a proxy for consumers’ knowledge about the product purchased. To this end, five-point Likert scales were employed, assigning point 1 to “strongly disagree” and 5 to “strongly agree”. Table 1 reports the summary statistics and a description of the data collected on both the products and consumers’ characteristics. The data collection of was carried out between March and July 2017 and consumers were recruited from a regionally representative sample of 14 hypermarkets and supermarkets, all located in the Apulian region (Italy). The sample of hypermarkets and supermarkets included at least one outlet in each of the six provincial capitals of the region, as well as a selection of the leading retailers in the Apulian region, namely Auchan, Conad, COOP, and Famila. 

### 2.2. Model and Statistical Analysis

To measure the monetary value of the product’s features, we used the standard hedonic price model first introduced by Rosen in 1974 [14]. According to hedonic price theory, a product is considered as a bundle of attributes. Each consumer in the market selects the set of features which maximizes his/her utility, subject to a budget constraint. Likewise, manufacturers maximize profits by setting the product’s price according to its attributes [14]. In a market for products presenting a unique bundle of attributes, buyers’ marginal bids and sellers’ marginal offers match at equilibrium and the joint envelope of consumers’ bids and sellers’ offers generate the hedonic price function [13]. Thus, the price, P, of a product, j, can be described as:

P_j_=f(Z_j_) (1)

where Z is a vector of product attributes belonging to product j and f (.) is an unspecified functional form. Equation (1) implies that the price consumers pay for product P is a function of the marginal monetary values of j’s attributes Z [14,15,16] and can be obtained by partially differentiating (1) with respect to each attribute. Furthermore, the implicit marginal price a consumer pays for the attribute Z corresponds to the marginal cost which the producer incurs in offering that attribute on the market. Equation (1) was estimated by ordinary least squares (OLS). 

Then, to measure the share of consumers able to correctly identify the origin of the EVOO products they purchased and infer the effectiveness of the mandatory COO information in correctly orienting consumers’ food choices, a cross-tabulation analysis was performed. A Pearson Chi-square test and a Goodman and Kruskal’s gamma statistic were used to assess whether the outcomes from the cross-tabulation were statistically significant, so testing the presence of a positive and statistically significant association between consumers’ statements about the origins of the product and the verified origins [17,18]. The work ends by profiling consumer groups based on their ability to correctly discriminate the product’s origins. Consumers’ socioeconomic characteristics, their interest in the information on the product’s label, in the origin of the product, as well as in branded products, were used to profile consumer groups. A Tukey test assessed whether consumers differ according to the characteristics listed above [19].

## 3. Results and Discussion

The estimated parameters of Equation (1), using the logarithmic transformation of the price as the dependent variable, are reported in the first column of Table 2, along with their standard errors in parenthesis. The functional form using the log-linear transformation of the price shows the lowest value of log-likelihood function testing it competitively with the linear and the box-cox transformation of the dependent variable. Marginal prices of each attribute (in percentage terms) are also calculated using Kennedy’s (1981) adjustment and reported in the last column [20] In Equation (1), we also control for brand fixed effects. For the sake of brevity, the resulting coefficients are not reported in the manuscript, but are available upon request. The baseline product is a non-organic EVOO from EU countries, filtered, and sold in 1 L glass bottle at an average price of 6.08 €/L. Based on the Ramsey’s RESET statistics for omitted variable bias [21], the model does not suffer from misspecification, and, since the null hypothesis of homoscedasticity of the Breusch–Pagan/Cook–Weisberg test cannot be rejected, the error terms are homoscedastic [22,23]. Skewness and Kurtosis test indicates the normality of the error terms distribution [24]. The model shows an adjusted R^2^ of 0.9694 and a statistically significant value of the F-statistic, suggesting the join significance of coefficients regressors. These statistics confirm that the semi-logarithmic specification of Equation (1) is the most appropriate among the possible functional forms for f (.).

The first notable finding reported in Table 2 is that the “Italian” origin label has a positive and significant effect on the price of EVOO sold in Italy and amounts to a price premium of +35%, relative to the baseline product, which is equivalent to +2.18€/liter. This result is consistent with other studies that found a willingness of Italian consumers to pay more for domestic EVOO products. Compared to the price of a European EVOO, “Non-European” EVOO is instead sold at a discount of −10.8%, equivalent to −0.70€/liter [6,7,8].

The geographical indication labels “GIs” show a positive and significant effect on price of +7.12 €/liter, or +112%, relative to the baseline product’s price. This attribute records the highest price premium among all the considered EVOO’s attributes and the result is consistent with several studies which also found that consumers, including those on the Italian market, prefer GIs products over regular ones and are willing to pay higher prices for such products [25,26,27]. The higher price of EVOO with GIs may, however, reflect the higher cost of GIs products, since farmers/producers seeking to sell their products with a GI label have to meet costly production standards that are frequently regarded as a barrier for the compliance with GIs standards [28,29,30].

Interestingly, the “Organic” attribute records a positive and significant impact on the EVOO price of +15.1% over the baseline product price, equivalent to a price premium of 0.91€/liter. The premium price associated with this attribute is likely to be the result of consumers’ willingness to pay for a “sustainable” product, which has been reported in several studies [31,32,33]. Products labeled as organic also are often perceived as healthier than regular ones, and, indeed, consumers’ primary reason for buying organic foods is their belief that these products support human health [34]. Thus, the premium price attached to organic EVOO may also be due to consumers’ willingness to buy products which they regard as supporting their health.

The marginal prices associated with the unfiltered attribute, “Unfiltered”, is positive and statistically significant. Unfiltered EVOO is sold with a markup of +21.6% or +1.28 €/liter. This suggests that the “unfiltered” claim on the label can be used by consumers to infer a higher degree of wholesomeness/naturalness [34], and a premium is thus associated with this attribute. 

The marginal prices associated with the packaging variables show positive and statistically significant coefficients. They indicate that premium prices are associated with EVOO products sold in glass packages smaller than 1 L. Products sold in glass bottles of 0.75 L (“Package Size 0.75 L”) benefit from a premium price of 0.912 €/liter, while products sold in 0.5 L glass bottles (“Package Size 0.5 L”) secure a premium price of 0.742 €/liter. The estimated marginal prices for products sold in other packaging material (“Other Packaging Material”), such as plastic or tin, as well as sold on promotion (“Promotion”) are negative, but not statistically significant.

With regard to Table 3, the data on consumers’ reports at checkout shows that 13.1% of EVOO consumers (129) in our sample were not aware of the origin of the product purchased, while a minority of 0.2% (2) reports their having purchased non-European EVOO. A larger share of EVOO consumers in the sample, approximately 28.0% (275), report having purchased a European EVOO and the majority of them, 255 out of 275, correctly identified the product’s origins. The largest group of EVOO consumers interviewed, 58.7% of the total sample (576), reports the purchase of Italian EVOO. In the latter group, one out of three consumers incorrectly identified the Italian product’s origin since they believed that they had purchased an Italian EVOO, but the product purchased actually was a blend of European EVOOs (165 out 576). The misuse about the product COO label then occurs more often when consumers report their having purchased Italian EVOO. 

A potential cause of the erroneous consumers’ identification of foreign EVOO as Italian may be related to the fact that consumers may be not aware of, or may not use, the COO information on the label. Another cause can be related to the fact that many non-Italian companies, after multiple mergers and acquisitions, hold in their portfolio several Italian EVOO brands (e.g., Bertolli, Carapelli, Sasso owned by Deoleo S.A.; Sagra and Filippo Berio by the Bright Food Group Co Ltd.). Companies use these brands to market non-Italian EVOO, so increasing the likelihood that consumers mistakenly infer from the brand name that the origin of the EVOO purchased is Italian. The use of a brand name with a more favorable image (in this case an Italian brand-name) to deliberately lead consumers to associate the origins of brand and product is a strategy previously reported for many other consumers goods markets [35]. If consumers use the brand as a clue to the origin of the product and other information on the origin of the product is not taken into account in the purchasing process, this marketing strategy lowers the ability of consumers to correctly identify the product’s origins. As proposed by Zhou, Yang, and Hui (2010, p. 204), *“the origin information for most brands may not be readily accessible either because global marketers have the desire to mask the origins of their brands or the globalization of firms and the cross-border acquisition of brands complicate the nature of brand origin”* [36].) Lastly, another hurdle for consumers in correctly identifying the origin of the product may be the fact that several Italian EVOO producers, aiming to offer consumers a greater variety of products, sell both Italian and non-Italian products under the same brand name. Such choice may further lower consumers’ ability to correctly identify the country of origin (COO) of the product during their food purchase.

Overall, the data in Table 3, reported in bold, indicates that 666 consumers, 67.8% of the sample, correctly associate the COO of the EVOO purchased. The positive association between the reported and verified origins of the product, and thus the overall ability to correctly associate the product and its origins, is statistically significant. The Goodman and Kruskal’s gamma statistic and the Pearson Chi-square test have a low *p*-values (<0.05). This indicates that the likelihood that consumers’ identification of the product’s origin corresponds to the correct one is high [17,18]. 

On the one hand, the results discussed above indicate that, overall, the mandatory COO information on EVOO products (EU Reg. 29/2012) is an effective tool in guiding consumers in the identification and selection of EVOO based on its origins. On the other hand, results show that there is still a consistent number of Italian consumers (187) who do not correctly associate the product with its actual origins and in most of the cases they are consumers who reported, and believed that they had purchased an Italian EVOO. Thus, the misuse of COO label mostly occurs where foreign EVOO products are identified as Italian.

Lastly, Table 4 identifies consumer groups according to their ability to correctly associate the COO of the EVOO purchased, characterizing them in relation to their socio-economic characteristics, as well as their interest in labeling information, in the origin of the product and in branded products more generally. 

The first consumer group, reporting that they are unaware of the product’s origin, encompasses 13.1% of consumers sampled. These consumers are mostly male, with a lower level of education compared to the average level of EVOO consumers in our sample. Furthermore, consumers unaware of the origins of the product live in a household with less than 3 individuals and their income is lower than in the other groups. These consumers purchase EVOO more often than others when it is sold on promotion at an average price of approximately 5.50 €/liter. Compared to consumers in the other two groups, these consumers also reported less interest in the information on the label, in the product’s origins and in brands.

The second group, representing 19.04% of EVOO consumers in the sample, encompasses individuals who incorrectly identify the country of origin of the product at the supermarket checkout. Consumers in this group are mostly male and have a higher level of education than those reporting unawareness of the product’s origins. Furthermore, they live in larger households and purchase products on promotion at 5.88 €/liter, which is not statistically different to the 5.50 €/liter paid by consumers that are not aware of the origins of the EVOO purchased. Compared to the previous group, consumers in this group report having a slightly greater interest in branded products.

The third and last consumer group, accounting for 67.82% of the total sample, encompasses consumers who correctly identify the product’s country of origin. This consumer group is largely composed of female consumers and has the highest level of education. These consumers purchase EVOO on promotion less frequently and also report their being highly interested in labeling information, as well as in the product’s origins and in branded products. On average, they pay 6.24 € for a liter of EVOO, a price that is higher and statistically different from that paid by consumers belonging to the two groups discussed before. 

Focusing on consumers who correctly identify Italian EVOO, reported in the last column of Table 4, they are again female EVOO shoppers with a higher level of education, highly interested in labeling information, in the origins of the product, and in branded products. This consumer group pays on average above 6.60 €/liter for an EVOO product. The data in Table 4 shows that consumers who place importance on information reported on the food label, including brand and the origins of the product, are more likely than others to correctly identify the product’s origins, if their level of education is higher than the average. 

Table 4 thus highlights how gender and education is likely to play a role in consumers’ ability to identify the origin of the EVOO product. This is consistent with findings from the general psychological theory about consumers and food labels, which identifies female and educated consumers as having greater ability to understand labeling information, as well as being more likely to take informed food choices and pay a higher price for their purchases [37,38].

## 4. Conclusions 

The present study confirms that the COO label is an effective tool to differentiate food products, in this case EVOO. In particular, our findings point out that, in Italy, the EVOO with domestic origin gains a premium price equal to +35% (+2.13 €/liter) compared to a product labeled as a blend of European EVOOs. Thus, on average, the mandatory COO labeling regulation for EVOO (Reg. (EU) No 29/2012) can be an effective tool for consumers to identify the origin of the product and for producers to differentiate their products. There is, however, a share of consumers in our sample, 19.04% (187), that incorrectly identifies the origin of EVOO purchased and this more often occurs among consumers who report having purchased Italian EVOO. On the one hand, this is likely due to producers’ branding strategies, which may hinder the effectiveness of COO information on labels in signaling the origin of the product. On the other hand, COO information, while useful for legal purposes, is not necessarily relevant to all consumers of EVOO, since other extrinsic clues like price may sometimes prevail in orienting EVOO choices.

Findings also indicate that education likely plays a role in correctly identifying the origin of the product, since it enhances consumers’ ability to process the information reported on the label of the product, including information about the product’s origins. Regarding this last point, government bodies, as well as food manufacturers and retailers, could implement signpost colored labeling to more easily communicate the origins of the product. A simple visual symbol to indicate a product’s origins may lower the cognitive effort needed to process the information on the label and so facilitate consumers’ identification of the country of origin. The use of a visual, color-based symbol has already been identified as a promising policy tool to support consumers in making healthier food choices at the supermarket. Several studies are offering encouraging findings in support of color-based labels as an effective policy approach to guiding consumers’ food purchases [39,40].

The present analysis is not, however, free from limitations. First, it does not explain the mechanism preventing consumers from decoding the origin of the product. This can depend on several additional psychological factors as well as on consumer knowledge related aspects, including the individual olive oil knowledge, which are not captured in the present analysis. Second, the study focuses on a consumer sample interviewed in a single Italian region, and on a single product category, EVOO, to test consumers’ ability to correctly identify a product’s origin. Future research will therefore aim to address these limitations by exploring the psychological mechanism underlying the incorrect association between the product and country of origin as well as exploring to what extent the olive oil knowledge affects such relation. Furthermore, we will expand the list of products against which consumer’s ability to correctly identify the country of origin is tested. 

## Figures and Tables

**Table 1:** Summary statistics related to product and consumer characteristics (982 observations).

Variables	Description	Mean
		(St. Dev.) ^a^
**Product variables**		
Dependent Variable		
*Price*	EVOO price €/l	6.088
		(1.7139)
Explanatory Variable		
*Organic*	1 = EVOO made from organic agricultural practices	0.024
*GIs*	1 = EVOO sold with Geographical Indications (DOP/PGI)	0.038
	
*Italian Origin*	1 = Italian EVOO	0.430
*Non-European Origin*	1 = Product from Non-European EVOOs	0.321
*European Origin*	1 = Product from European EVOOs	0.249
*Filtered*	1 = Filtered Product	0.989
*Unfiltered*	1 = Unfiltered Product	0.011
*Product in Promotion*	1 = Product sold in promotion	0.563
*Glass Packaging*	1 = Product sold in glass packages	0.986
*Other Packaging Material*	1 = Product sold in other packages (e.g., plastic)	0.014
*1 L*	1 = Package Size 1liter	0.891
*0.75 L*	1 = Package Size 0.75 L	0.078
*0.5 L*	1 = Package Size 0.5 L	0.031
**Consumer variables**		
*Gender*	1=Female consumer	0.529
*Household size*	1–6 = Number of household members	3.089
		(1.1311)
*Child below 18 years old*	1 = Household with child below 18 years old	0.671
*Education*	1–3 = 1 Middle school education, 2 High school education, 3 University education	2.227
		(0.6935)
*Income*	1–3 = 1 Individual monthly income below 1000€,	2.258
	2 between 1001€–2000€, 3 above 2001€	(0.6860)
*Shopping EVOO on offer*	1 = Purchase an EVOO on promotion	0.562
*Interest in label information*	1–5 = “I am interested in labeling”, where 1 stands for “strongly disagree” and 5 for “strongly agree”	4.220 (0.7075)
*Interest in product origin*	1–5 = “I am interested in product origins”, where 1 stands for “strongly disagree” and 5 for “strongly agree”	3.910
		(0.9470)

*Interest in branded products*	1–5 = “I am interested in branded products”, where 1 stands for “strongly disagree” and 5 for “strongly agree”	3.899
		(0.9662)


^a^ For all binary variables the mean represents the percentage of observations, the value of the standard deviation is omitted.

**Table 2:** Estimated parameters and percentage of premium price.

Variable	*β*	Percentage, Premium Price ^a^
*Organic*	0.141 **	+15.1
	(0.0710)	
*GIs*	0.776 ***	+117.0
	(0.0570)	
*Italian Origin*	0.301 ***	+35.0
	(0.0830)	
*Non-European Origin*	−0.114 ***	−10.8
	(0.0210)	
*Unfiltered*	+0.245 **	+21.6
	(0.1030)	
*Product in promotion*	−0.001	−0.1
	(0.0060)	
*Other Packaging Material*	−0.123	−11.5
	(0.1020)	
*0.75 L Package Size*	0.143 ***	+15.4
	(0.0450)	
*0.5 L Package Size*	0.115 *	+12.2
	(0.0630)	
*Constant*	1.726 ***	
	(0.0970)	
Number of Observations	982	
R-square	0.961	
*Specification test*		
Ramsey’s RESET F(2, 870)	1.32	
*p*-value	0.2672	
*Heteroskedasticity*		
Breusch-Pagan/Cook-Weisberg χ^2^(1)	0.18	
*p*-value	0.6731	
*Normality*		
Skewness and Kurtosis χ^2^(2)	0.1355	
*p*-value	0.125	

^a^ Adjustment made according to Kennedy (1981). *, ** and *** are 10, 5 and 1 percent significance levels.

**Table 3:** Consumer-declared origin of the EVOO products purchased and the actual one.

EVOO’s Origin of Actual Purchases	Non-European Countries	European Countries	Italian	Total Respondents ^a^
EVOO’s origin declared:				
“*I am unaware of the origin of the EVOO I purchased*”	1 (100%)	112 (20.97%)	16 (3.58%)	129 (13.1%)
“*It is a blend of Non-European EVOOs*”	0 (0.00)	2 (0.37%)	0 (0.00)	2 (0.20%)
“*It is a blend of European EVOOs*”	0 (0.00)	**255** **(47.75%)**	20 (4.47%)	275 (28.0%)
“*It is 100% Italian EVOO*”	0 (0.00)	165 (30.90%)	**411** **(91.95%)**	576 (58.7%)
Total respondents	1	519	462	982
Pearson χ^2^(6) = 373.7349 Pr = 0.000; Gamma = 0.6986 ASE = 0.036				

^a^ The share of respondents over the total number of respondents of the column is reported in parenthesis. The number of respondents who correctly indicated the product’s origin is in the highlighted in bold.

**Table 4:** Characteristics of consumers groups.

	Full Sample (Obs. = 982)			Subsample (Obs. = 576)	
	Unaware of the Origin of the EVOO	Incorrectly Classify the EVOO Origin.	Correctly State the EVOO Origin.	Incorrectly Classify the Italian EVOO Origin.	Correctly Classify the Italian EVOO Origin.
Share of respondents	13.10%	19.04%	67.82%	28.64%	71.36%
**Consumer variables**					
*Gender*	0.380 ^a^	0.460 ^a^	0.577 ^b^	0.461 ^a^	0.604 ^b^
	(0.4872)	(0.4997)	(0.4945)	(0.5003)	(0.4896)
*Education*	1.791 ^a^	1.930 ^b^	2.332 ^c^	1.985 ^a^	2.360 ^b^
	(0.6334)	(0.7336)	(0.6645)	(0.7170)	(0.6619)
*Household size*	2.783 ^a^	3.166 ^b^	3.092 ^b^	3.156 ^a^	3.060 ^a^
	(1.1247)	(1.2134)	(1.0992)	(1.1667)	(1.1016)
*Child less than 18Y*	0.426 ^a^	0.615 ^ab^	0.716 ^b^	0.602 ^a^	0.680 ^a^
	(0.6821)	(0.8369)	(0.8128)	(0.8270)	(0.8046)
*Income*	2.031 ^a^	2.241 ^ab^	2.251 ^b^	2.283 ^a^	2.367 ^a^
	(0.7340)	(0.6727)	(0.6888)	(0.6473)	(0.6558)
*Purchase on promotion*	0.605 ^a^	0.572 ^a^	0.520 ^b^	0.680 ^a^	0.375 ^b^
	(0.4908)	(0.4961)	(0.5000)	(0.4678)	(0.4848)
*Interest in label information*	3.860 ^a^	4.305 ^ab^	4.255 ^b^	4.248 ^a^	4.405 ^b^
	(0.9499)	(0.6625)	(0.6812)	(0.6564)	(0.6430)
*Interest in product origin*	3.829 ^a^	3.947 ^ab^	3.983 ^b^	3.829 ^a^	4.244 ^b^
	(1.0978)	(0.9601)	(0.9134)	(0.9633)	(0.8518)
*Interest in branded products*	3.605 ^a^	3.952 ^b^	3.959 ^b^	3.879 ^a^	4.103 ^b^
	(1.0998)	(0.9798)	(0.9354)	(0.9671)	(0.9084)
*Price*	5.489 ^a^	5.881 ^ab^	6.241 ^b^	5.740 ^a^	6.630 ^b^
	(1.0329)	(1.5327)	(1.8203)	(1.5083)	(2.0618)

a, b, c: values with the same letter as the superscript indicate no statistically significant differences between the groups (columns) based on the pairwise mean comparison across groups using Tukey Kramer test, *p* < 0.05.
